# Identification of Relevant Sociocognitive Determinants Explaining Multiple Parental Sun Protection Behaviors

**DOI:** 10.1177/10901981211010434

**Published:** 2021-05-31

**Authors:** Karlijn Thoonen, Liesbeth van Osch, Rik Crutzen, Hein de Vries, Francine Schneider

**Affiliations:** 1 Maastricht University, Maastricht, the Netherlands

**Keywords:** children’s sun safety, parental behavior, primary prevention, skin cancer, sun protection

## Abstract

**Background:**

Adequate sun safety during childhood is crucial for decreasing skin cancer risk in later life. Although parents are an essential target group in applying sun protection measures for their children, insight into the determinants associated with their sun protection behaviors is limited.

**Aims:**

This study aims to identify the most relevant determinants in predicting multiple parental sun protection intentions and behaviors in different sun exposure situations.

**Method:**

A longitudinal survey study with two measurements was conducted among Dutch parents (*N* = 670) of children (4–12 years old). Twenty-seven sociocognitive determinants were examined in terms of relevance regarding four parental sun protection behaviors in different sun exposure situations. The Confidence Interval-Based Estimation of Relevance approach was used to visualize room for improvement (sample means) on all determinants and their association strengths (correlations) with sun protection intentions and behaviors.

**Results:**

Behavior-specific rather than generic determinants were most relevant in explaining all sun protection behaviors. Of these determinants, attitude, self-efficacy and action planning, and especially parental feelings of difficulty in performing sun protection behaviors, were most relevant. Altogether, the explained variance of all sociocognitive determinants was highest for shade-seeking behavior (*R*^2^ = .41 and .43) and lowest for supportive behavior (*R*^2^ = .19 and .29) in both planned and incidental sun exposure situations, respectively.

**Discussion:**

This study provides detailed insight into relevant sociocognitive determinants of parental sun protection behaviors in various sun exposure situations and directions for composing parental skin cancer prevention interventions.

**Conclusions:**

Future composition of sun safety interventions should emphasize on enhancing parental feelings of self-efficacy, especially for shade-seeking and clothing behaviors.

Skin cancer incidence, and especially melanoma, is rising excessively worldwide (Apalla et al., 2017). The burden of melanoma is highest in Europe, North America, and Oceania, where together 84% of incidences in 2018 occurred (Bray et al., 2018). Incidence rates are expected to rise even further over the coming decades in fair-skinned populations (Whiteman et al., 2016), emphasizing the importance of prevention efforts. Exposure to ultraviolet radiation (UVR) and sunburn incidence, particularly during early childhood, are the most important risk factors in the etiology of melanoma (Arnold et al., 2018; Whiteman et al., 2001). Globally, one to two thirds of children experience at least one sunburn every year (Ackermann et al., 2016; Behrens et al., 2013; Day et al., 2017; Hall et al., 2001; O’Riordan et al., 2003; Wu et al., 2019). Therefore, it is warranted to reduce the amount of received UVR and sunburn occurrence by performing adequate sun protection behaviors (e.g., using sunscreen, seeking shade) in children, specifically.

Parents play a crucial role in directly or indirectly protecting their children from overexposure to UVR and sunburn. Young children generally rely on their parent’s sun protection behavior toward them (Thoonen et al., 2019). As they grow older, parents serve as their primary role models from which children learn how to perform these behaviors themselves (Hagger & Hamilton, 2019). Moreover, studies investigating the parental role in sun protection reveal that parents are important gatekeepers in encouraging children’s own sun protection attitudes and behaviors (Dobbinson et al., 2012; O’Riordan et al., 2003; Turrisi et al., 2007). Parents are therefore essential targets to promote sun protection in children.

Although guidelines exist to adequately protect one’s skin against UVR (Watson et al., 2014), reported parental adherence varies among studies. For example, studies report that only 17% of parents perform sun protection among their children correctly (Tan et al., 2018), or 75% of parents inadequately apply sunscreen to their child (Klostermann et al., 2014), while other studies describe high parental performance of sun protection behaviors (Gefeller et al., 2016; Stanton et al., 2004). Sun protection ideally comprises simultaneous application of multiple protective measures (Watson et al., 2014), with seeking shade and wearing protective clothing increasingly being recommended. However, sunscreen use is often the most preferred, and regarded as the safest, precaution taken by the general population (Koch et al., 2017; Linos et al., 2011) and among parents (Klostermann et al., 2014; Thoonen et al., 2019). Nonetheless, an overreliance on sunscreen can occur, resulting in an increased risk of unprotected UVR exposure and sunburn (Autier, 2009; Autier et al., 2007; Ghiasvand et al., 2015). This sunscreen paradox is particularly perceived during planned sun exposure (e.g., when going to the beach). However, results may differ for situations in which people are not purposely exposed to sun (e.g., when walking or bicycling; Autier, 2009). Hence, sun protection approaches need to address comprehensive sun protection behaviors in various situations (Sinclair & Foley, 2009).

Understanding why parents engage in sun protection behaviors by examining relevant sociocognitive determinants is fundamental to address these determinants in future interventions (Kok et al., 2016). Various studies have investigated the role of sociocognitive determinants (e.g., attitude, social norms) in the onset of sun protection behaviors among adults (Bränström et al., 2004; Myers & Horswill, 2006; White et al., 2015), but comprehensive studies investigating parent-for-child sun protection behaviors are limited. These studies often focus on premotivational determinants such as knowledge or risk perceptions (de Vries et al., 2012; Li et al., 2011), or attitude (Cercato et al., 2013; Johnson et al., 2001; Li et al., 2011); include parents of very young (2 to 6 years old) children (Hamilton et al., 2017; Thomson et al., 2012; Turner & Mermelstein, 2005); or have sunscreen use as main outcome (Hamilton et al., 2017; van Osch, Reubsaet, Lechner, Candel, et al., 2008). Although preliminary, results of these studies indicate an influential role of sociocognitive determinants such as anticipated regret, attitude, self-efficacy, and action planning in parental sun protection behaviors. To target sun safety interventions for parents, identification of the most relevant determinants foregoing various parental sun protection behaviors is imperative (Bartholomew Eldredge et al., 2016; Hamilton et al., 2017). Gaining comprehensive insight by investigating the relevance of both generic (e.g., knowledge about skin cancer) and behavior-specific (e.g., attitude toward clothing behavior) sociocognitive determinants is warranted.

Despite the importance of sun safety during childhood and the powerful parental role in initiating sun protection behaviors (Dobbinson et al., 2012; Thoonen et al., 2019), targeted interventions are so far restricted. Moreover, those interventions targeted at parents report limited effects on parental behavior, such as clothing or seeking shade (Bellamy, 2005; Gritz et al., 2005; Hart & DeMarco, 2008; Hunter et al., 2010; Rodrigues et al., 2013). This is also illustrated by systematic reviews concerning effectiveness of educational interventions, revealing that conclusions about the effectiveness regarding parent-for-child sun protection behaviors could not be drawn (Saraiya et al., 2004), reported limited efficacy (Bellamy, 2005) or did not specifically report on parental target groups (Rodrigues et al., 2013). A recent review discussed the limitation of available evidence among children’s caregivers when assessing the effectiveness of community-wide sun safety interventions (Sandhu et al., 2016). Although some positive effects of educational interventions on parental sun protection behaviors are reported, strong conclusions about the effectiveness of these parent-for-child interventions remain absent due to lack of data.

This study aims to identify most relevant generic and behavior-specific sociocognitive determinants regarding their room for improvement and association with both direct (i.e., sunscreen use, clothing, seeking shade) and indirect (supporting the child in performing sun protection measures) parent-for-child sun protection behaviors. Furthermore, this study distinguishes relevance of sociocognitive determinants in both planned (e.g., going to the beach) and incidental (e.g., bicycling) sun exposure situations.

## Method

### Study Design

Data from a longitudinal cohort study regarding parental sun protection with a total of four measurements were used. For this study specifically, data from the second (October 2016 [T1]) and third (October 2017 [T2]) measurement were analyzed, as all determinants (T1) and behaviors (T2) relevant for this study aim were included. This study was exempted from approval by a medical ethics committee, since participants were not exposed to medical procedures or behavioral demands (Central Committee on Research Involving Human Subjects, 2002). The data collected in this study were pseudonymized, meaning that the research team could not identify specific persons within the dataset (Crutzen et al., 2019). STROBE guidelines for observational research were followed to report this study (Von Elm et al., 2007).

### Participants and Recruitment

The Dutch research organization TNS-KANTAR (2019) invited an eligible sample of parents who were members of an existing research panel, representative of the Dutch general population based on education and income. Parents were eligible for participation if they had at least one child in the primary school age. Online informed consent was obtained by TNS-KANTAR (2019). The sample of parents received one invitational email and one reminder per measurement. In these invitations, a direct link was provided to the online questionnaires assessing direct (i.e., sunscreen use, clothing, and seeking shade) and indirect (supportive) sun protection behaviors and related behavioral determinants. Parents were asked to answer the questions regarding the same child (the youngest in their household) during both measurements. After completion of each questionnaire, parents received a small incentive consisting of gift vouchers.

### Measurement

The online survey assessed the following aspects: (1) demographic characteristics, (2) execution of sun protection behaviors, (3) generic determinants, and (4) behavior-specific determinants. Sections 3 and 4 were based on the premotivational, motivational, and postmotivational phases of the I-change model (de Vries, 2017), an integrative theoretical framework for understanding health behavior.

#### Demographic Characteristics

Age, gender, and educational level of parents were assessed, together with age and gender of their child. Educational level of parents was categorized as low (1; e.g., primary education)/medium (2; e.g., secondary vocational education)/high (3; e.g. university education), conform guidelines of Statistics Netherlands (Nuffic, 2019; Statistics Netherlands [CBS], 2016). Children’s age was classified into three groups (4 to 6 years, 7 to 9 years, and >10 years; cf. Dutch primary school system).

#### Direct and Indirect Sun Protection Behaviors

Direct sun protection behaviors consisted of (1) applying sunscreen, (2) providing the child with UV-protective clothing and/or garments, and (3) seeking shade. Indirect behavior consisted of supporting the child in conducting his or her own sun protection behaviors (defined as advising, facilitating sun protection behaviors, and checking/monitoring whether the child applied sun protection behaviors). The frequency of self-reported application of these behaviors was assessed regarding the past summer season, using a 5-point Likert-type scale ranging from *never* (1) to *always* (5). A nonapplicable answer category was included for indirect behavior, in case the child was too young to be supported in his or her own sun protection behavior.

Execution of all sun protection behaviors was assessed for two different types of sun exposure. First, *planned sun exposure* (PS), consisting of situations during which parents and/or their child expected and intended to be exposed to the sun (e.g., going to the swimming pool or beach) and, second, *incidental sun exposure* (IS) comprised situations of unintentional sun exposure (e.g., bicycling or playing outside).

In total, eight outcome measures (three direct behaviors and one indirect behavior; all in two situations) were assessed. To clarify the distinction of behaviors and situations, parents received explanation about the separate sun protection behaviors beforehand, according to guidelines from the Dutch Cancer Society (KWF Kankerbestrijding, 2020) and examples of different sun exposure situations (https://osf.io/vwr2g/).

#### Generic Sociocognitive Determinants

Generic sociocognitive determinants were assessed universally across all four behaviors. The construct *knowledge* consisted of 14 true-false statements regarding UVR exposure, sunburn, and skin cancer (*correct* [1], *incorrect* [0], or *I don’t know* [0]). *Risk perception* consisted of 12 questions addressing cognitive (four items) and affective (four items) risk susceptibility, and severity (four items) concerning both sunburn and skin cancer, addressing PS and IS situations. *Anticipated regret* contained four questions regarding regret parents feel when their child would experience a sunburn. Furthermore, the frequency of children’s sunburn during both the previous summer season and across their lifetime was assessed as an indicator for cues to action. Last, parent’s *attitude* toward the importance of their children’s tanned skin was assessed. All items were assessed using a 5-point Likert-type scale.

#### Behavior-Specific Determinants

Behavior-specific sociocognitive determinants were assessed for each separate sun protection behavior, in which two questions, regarding PS and IS situations, were assessed per item. For every sun protection behavior, attitude was measured by two items assessing the extent to which parents regarded the sun protection behavior as important or unimportant, as well as pleasant or unpleasant. Social norm was measured by two items per behavior, distinguishing the perceived norms based on the opinion of partners (if applicable) and important others. Self-efficacy was measured by two items per behavior, which differentiated parental experiences of difficulty and ability of performing sun protection behaviors. Action planning was measured by one item per behavior, assessing whether a specific plan was formulated to perform sun protection. Last, intention toward each specific sun protection behavior was assessed by one item (de Vries, 2017). Table 1 provides a set of exemplary items.

**Table 1.** Exemplary Items of Behavior-Specific Determinants Concerning Sunscreen Use in Incidental Situations.^a^

Determinants	Subconcepts	Items	Answer categories and coding
Attitude			
Importance	Importance of sunscreen use	When my child is engaging in *outdoor activities* (e.g., playing, bicycling) on sunny days, I think that adequately applying sunscreen to my child is [ . . . ]	1 = *not important*, 2 = *slightly important*, 3 = *moderately important*, 4 = *important*, 5 = *very important*
Pleasantness	Pleasantness of sunscreen use	When my child is engaging in *outdoor activities* such as playing, exercising, bicycling, or walking on a sunny day, I think adequate sunscreen use for my child is [ . . . ]	1 = *not pleasant*, 2 = *slightly pleasant*, 3 = *moderately pleasant*, 4 = *pleasant*, 5 = *very pleasant*
Social norm			
Partner	Partner’s opinion about sunscreen use	When my child is engaging in *outdoor activities* such as playing, exercising, bicycling, or walking on sunny days, *my partner* thinks it is important that we adequately use sunscreen for our child.	1 = *totally disagree*, 2 = *disagree*, 3 = *neutral*, 4 = agree, 5 = totally agree, 6 = not applicable (= 99)
Important others	Opinion of important others about sunscreen use	When my child is engaging in *outdoor activities* such as playing, exercising, bicycling, or walking on sunny days, *important people* around me think it is important that I/we adequately use sunscreen for my child.	1 = *totally disagree*, 2 = *disagree*, 3 = *neutral*, 4 = *agree*, 5 = *totally agree*
Self-efficacy			
Difficulty	Difficulty to apply sunscreen	When my child is engaging in *outdoor activities* (e.g., playing, bicycling) on sunny days, how *difficult* is it for you to make sure he/she is adequately protected with sunscreen?	1 = *very difficult*, 2 = *difficult*, 3 = *neutral*, 4 = *easy*, 5. *very easy*
Ability	Being able to apply sunscreen	If my child is engaging in *outdoor activities* (e.g., playing, bicycling) on sunny days, I am *able* to make sure he/she is adequately protected with sunscreen.	1 = *definitely not*, 2 = *probably not*, 3 = *neutral*, 4 = *probably*, 5 = *definitely*
Intention			
	Intention to apply sunscreen	When your child is engaging in *outdoor activities* (e.g., playing, bicycling) on sunny days, do you intend to adequately apply sunscreen to him/her?	1 = *definitely not*, 2 = *probably not*, 3 = *might*, 4 = *probably*, 5 = *definitely*
Action planning			
	Formulation of action plan(s) to apply sunscreen	When your child is engaging in *outdoor activities* (e.g., playing, bicycling) on sunny days, do you have a specific plan to adequately use sunscreen for him/her?	1 = *definitely not*, 2 = *probably not*, 3 = *might*, 4 = *probably would*, 5 = *definitely would*
Sun protection behavior			
	Parent-for-child sunscreen use during the previous summer season	When your child was engaging in *outdoor activities* such as playing, exercising, bicycling, or walking on a sunny day during the previous summer, to what extent did you adequately apply sunscreen to protect your child?	1 = *never*, 2 = *rarely*, 3 = *sometimes*, 4 = *often*, 5 = *very often*

a The full questionnaire can be retrieved from Open Science Framework (https://osf.io/vwr2g/).

### Analyses

Descriptive analyses were performed using IBM SPSS Statistics for Windows, Version 24.0 (IBM Corp, 2016). Not applicable answers to questions were excluded from analyses. A sum score of the number of correctly answered knowledge items was computed (ranging from 0 [low levels of knowledge] to 14 [high levels of knowledge]), which was then recoded into a scale ranging from 1 to 5 to enhance visual comparison between all determinants imputed in further analyses (as they were assessed using 5-point Likert-type scales). For the analyses, determinants from T1 (*n* = 28) and behavioral outcomes from T2 (*n* = 8) were used.

Confidence interval–based estimation of relevance (CIBER) was used to establish relevance of parental sociocognitive determinants regarding their sun protection behaviors (Crutzen et al., 2017). CIBER is a data visualization method integrating descriptive statistics that combine two types of analyses: assessing (1) univariate distribution of each determinant (based on means), and (2) associations with behavioral outcomes (based on correlations). Univariate distributions show the room for improvement regarding each determinant (i.e., how high participants score on the scale). This needs to be combined with the association with behavioral outcomes, as those determinants that are associated with behavior and where there is room for improvement are the most relevant candidate variables to intervene upon. For both means and correlations, confidence intervals show the accuracy with which these can be estimated. CIBER visualizes this information to facilitate comparison on spatial dimensions, which is necessary when making selections for intervention development. Furthermore, visualization foregoes the apparent accuracy and objectivity produced by numbers. Given the relative width of most sampling distributions and the subsequent variation that occurs in estimates over samples (Moinester & Gottfried, 2014; Peters & Crutzen, 2020), caution in basing decisions on the exact computed numbers seems prudent. CIBER plots were created using the *R* (R Core Team, 2017) package *behaviorchange* (Peters, 2018).

## Results

### Sample Characteristics

At T2, 670 parents remained (74.1% response rate; 58.5% mothers; 54.3% higher educated; mean income range: 69.000–82.300) and were included in the analyses. Attrition analyses indicated that demographic characteristics were not significantly associated with drop out on T1 and T2. From these parents, 339 (50.6%) and 331 (49.4%) answered the questionnaires regarding sun protection of their son and daughter, respectively. Children were aged between 4 and 14 years (modus = 6; *M* = 8.8; *SD* = 2.6). Self-reported sunburn occurred at least once among 29.1% of the children during the previous summer season (*M* = 1.3; *SD* = .5) and among 77.4% of the children throughout their lives (*M* = 1.9; *SD* = .6). With regard to direct sun protection behaviors, sunscreen was frequently (i.e., “often” and “always”) applied by parents in PS (88.2%) as well as in IS (64.8%) situations. Additionally, frequent execution of indirect behavior was performed by a majority of the parents in both PS and IS situations (77.0% and 68.8%, respectively).

### Relevance of Behavior-Specific Determinants

#### Direct Behaviors

Overall, both beliefs assessing *attitude* demonstrated highest sample means regarding sunscreen use in both PS and IS situations. Overall, the belief regarding importance demonstrated higher mean scores and therefore less room for improvement than the belief about pleasantness of the sun protection behaviors. Moreover, for all sun protection behaviors, both attitudinal determinants indicated strong positive associations with both sun protection intentions and behaviors in PS as well as IS situations.

With regard to *social norm*, sample means were again higher for sunscreen use than for clothing and shade-seeking behavior. The extent to which partners believe that sun protection is important demonstrated highest mean scores for all behaviors, whereas the extent of importance among other people depicted lower mean scores, implicating more room for improvement. For all sun protection behaviors, the importance of a partner’s opinion concerning the sun protection behavior demonstrated positive associations with sun protection intentions and behaviors for all three behaviors, in both PS and IS situations. Compared with other determinants, the importance of sun protection according to other people often indicated the lowest associations.

*Self-efficacy* demonstrated lowest sample means compared with other determinants, especially for clothing and shade-seeking behavior. Especially, feelings of difficulty depicted lowest scores across almost all behaviors, indicating high perceived difficulty to perform sun protection behaviors and notable room for improvement. Compared with other behaviors, parents indicated highest difficulty for seeking shade. Moreover, being able to perform sun protection behaviors depicted notable room for improvement as well. Both aspects of self-efficacy demonstrated highest positive associations with intentions and performance across all behaviors, with feelings of ability to perform sun protection behaviors showing most positive associations.

Last, formation of *action plans* demonstrated low sample means and therefore opportunity for improvement for all sun protection behaviors in both PS and IS situations, with again seeking shade indicating the lowest scores across behaviors. Following self-efficacy, action planning often depicted the second highest association with intentions and behaviors to perform sun protection behaviors.

**Figure 1.**
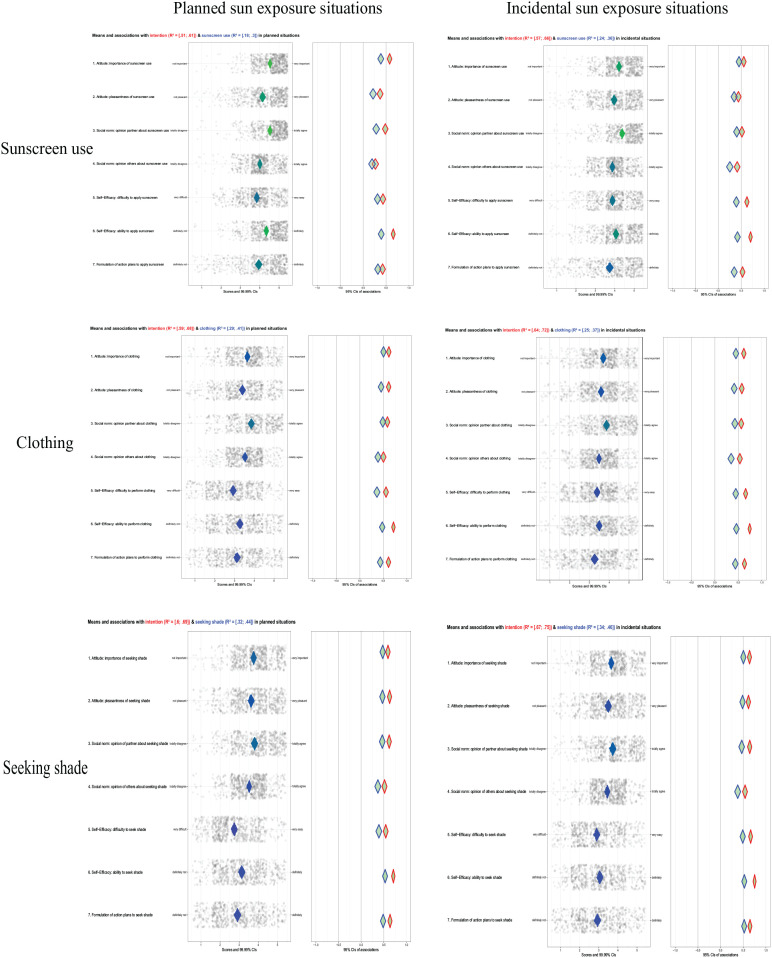
Confidence interval–based estimation of relevance (CIBER) plots for, respectively, sample means ranging from red (lower end of the scale), via blue (middle), to green (higher end of the scale) and associations of behavior-specific determinants with the intentions to perform sun protection behaviors (red outlined) and execution of sun protection behaviors (blue outlined) in planned (left) and incidental (right) situations.

In Figure 1, an overview of the relevance of behavior-specific determinants regarding direct sun protection behaviors is provided for both PS and IS situations.^1^

#### Indirect Behaviors

**Figure 2.**
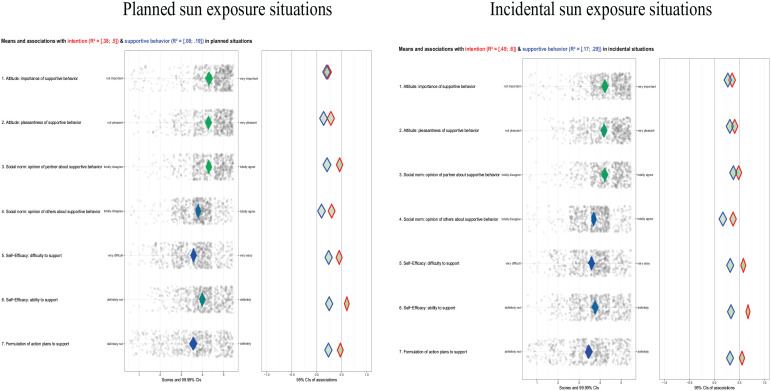
Confidence interval–based estimation of relevance (CIBER) plots for, respectively, sample means ranging from red (lower end of the scale), via blue (middle), to green (higher end of the scale) and associations of behavior-specific determinants with the intentions to support (red outlined) and execution of supportive behavior (blue outlined) in planned (left) and incidental (right) situations.

Overall, behavior-specific determinants foregoing supportive behavior depicted comparable sample means in PS and IS situations. Moreover, sample means were highest for attitude and social norm (concerning partner’s opinion) and lowest for self-efficacy (difficulty of providing support) and action planning. Associations with intentions and behaviors were most positive for determinants related to self-efficacy and action planning, with the self-efficacy belief about ability having the highest associations (consider Figure 2). Since mostly older aged children are being encouraged to perform sun protection behaviors themselves, a smaller sample of parents reported execution indirect behavior (*n* = 637; 95.1%).

### Relevance of Generic Determinants Across All Behaviors

*Knowledge* portrayed high sample mean scores across all behaviors, whereas sample means regarding *risk perception* depicted lower scores. Particularly, lowest scores were reported for cognitive and affective risk susceptibility concerning skin cancer in IS situations. Moreover, higher sample means were demonstrated for determinants concerning skin cancer severity, with the severity of skin cancer in comparison with other cancer types depicting lowest mean scores. *Anticipated regret* was moderate to high across all behaviors, in which regret concerning sunburn was notably lower than regret concerning skin cancer development. The group mean for the attitude concerning children’s *tanned skin* was low.

Associations of the generic determinants with intentions (*r* range .07–.37) and behaviors (*r* range .07–.34) were low and varied slightly across behaviors. Moreover, previous sunburn and the positive attitude toward children’s tanned skin were negatively associated with all sun protection intentions (*r* range −.08 to −.30) and behaviors (*r* range −.01 to −.30). Table 2 provides all sample distributions and associations with sun protection intentions and behaviors.

### Explained Variance Across All Behaviors

On average, the full set of sociocognitive determinants explained 19% to 41% of the variance in all sun protection behaviors in PS situations and 28% to 43% in IS situations, in which shade-seeking behavior demonstrated highest and supportive behavior demonstrated lowest explained variance. The average explained variance for intentions to perform sun protection behaviors ranged between 46% and 66% in PS, and 58% and 73% in IS situations.

## Discussion

This study provides detailed insight in relevant sociocognitive determinants for predicting parental sun protection behaviors in various sun exposure situations. Relevance of determinants was indicated by both room for improvement and their associations with sun protection intentions and behaviors. Overall, associations between generic determinants and sun protection intentions and behaviors were low, whereas behavior-specific determinants were highly associated with these intentions and behaviors. Moreover, attitude, self-efficacy, and action planning were particularly relevant regarding shade-seeking and clothing behaviors in both sun exposure situations. Additionally, determinants altogether showed greater relevance for explaining sun protection intentions and behaviors in incidental rather than PS situations as well as for shade-seeking and clothing behavior rather than for sunscreen use and indirect behavior.

Although the findings in this study clearly demonstrated the importance of behavior-specific determinants, current educational sun safety interventions predominantly target generic (e.g., knowledge and risk perception in general) instead of behavior-specific determinants, lacking evidence on long-term improved sun protection behaviors when directed at children (Bellamy, 2005; Buller & Borland, 1999; Saraiya et al., 2004) or at parents (Bellamy, 2005; Cercato et al., 2013; Rodrigue, 1996). Increasing knowledge and improving one’s health beliefs only is evidently not sufficient for establishing health behavior change (Glanz et al., 2015; Webb & Sheeran, 2006). With regard to skin cancer prevention specifically, skin cancer knowledge and awareness are not sufficient to establish sustainable sun protection behavior (Hart & DeMarco, 2008). Focusing on additional behavior-specific determinants in parental sun safety interventions is therefore highly recommended.

When thoroughly examining the relevance of specific sociocognitive determinants, a few findings emerged. First, attitudes supportive of sun protection among parents seem important to include in interventions. Although in this study the room for improvement of attitude was lower than for other determinants, associations with all sun protection intentions and behaviors were strong. Parental attitudes are important in predicting various parent-for-child behaviors (Hamilton et al., 2020) and appear to strongly influence children’s own attitudes with regard to sun protection (Stanton et al., 2004). Second, self-efficacy regarding execution of sun protection behaviors was found to be essential. The positive association between parental self-efficacy and sun protection toward their children has been demonstrated before (Hamilton et al., 2020; Tripp et al., 2013; Turner & Mermelstein, 2005). Besides, this study demonstrated the distinction between relevance of feelings of difficulty and ability. Notable room for improvement was especially shown regarding the experienced difficulty in performing sun protection behaviors. Investigating the reasons underlying of parental feelings of difficulty is essential for selecting specific behavior change methods for intervention development (Kok et al., 2016). Although the larger project in which this study was conducted indicated difficult situations to perform sun protection (e.g., when it is too hot to wear clothing or when a child rejects to wear sunglasses), strong conclusions cannot be drawn. Moreover, confusion concerning recommended sunscreen application (Robinson et al., 2000) or ambiguity about achieving sufficient vitamin D (Littlewood & Greenfield, 2018) could increase feelings of difficulty. Moreover, behavior-specific determinants were more strongly associated with sun protection intentions than behaviors, implying the well-documented intention–behavior gap (Sniehotta et al., 2005). The relevance of action planning for all sun protection behaviors reported in this study has been previously documented for parental sunscreen use (van Osch, Reubsaet, Lechner, & de Vries, 2008). After understanding the particular difficulties to perform sun protection behaviors more clearly, directions for formulating specific action and coping plans can be integrated in future interventions to increase the likelihood of behavior change (Bailey, 2019; Schwarzer, 2008).

This study also found negative associations between determinants and intentions and behaviors. Parents reporting on their child having previously experienced sunburn appear to subsequently apply less sun protection behaviors than parents whose child did not experience sunburn. Although the association between children’s previous sunburn and future sun protection behavior has not been thoroughly investigated (Champion & Skinner, 2008), studies found positive correlations, indicating that sunburn functions as a motivating factor for sun protection behavior (Littlewood & Greenfield, 2018; Robinson et al., 2000). However, trends in sunburn occurrence remaining high over time have been reported (Dusza et al., 2012; Ghiasvand et al., 2015). Since sunburn was assessed cumulatively in the current study, its negative association with sun protection behaviors could be caused by a behavioral pattern of noncompliance among parents. Furthermore, a negative association between a tan-favoring attitude and sun protection behaviors was apparent. Since the latter is corresponding with results in previous parental-focused studies (Gefeller et al., 2014; O’Riordan et al., 2003), parental beliefs concerning a tan appearing healthy or pretty should be targeted in interventions to enhance sun protection practices.

Overall, variance in sunscreen use was less explained by determinants than shade-seeking and clothing behaviors. Since a strong parental preference for applying sunscreen among their children is known (Stanton et al., 2004; Thoonen et al., 2019), this could imply that sunscreen use originates by recitation and therefore becomes a habitual rather than reasoned or deliberately controlled behavior (Neal et al., 2006). Since frequently performed behavior can increase skill acquisition and reduce the impact of sociocognitive determinants on intentions and behaviors (Aarts et al., 1998; Webb & Sheeran, 2006), behaviors can be triggered directly by certain cues in a situation in which the behavior was performed in the past (Orbell & Verplanken, 2010). Moreover, parents explicitly mentioned origination of habitual use of sun protection measures in situations where the behaviors were firstly established, such as going to the beach (Hamilton et al., 2016). This is also illustrated by the higher explained variance during IS situations in this study, indicating the more deliberately origination of sun protection behaviors in these situations. Understanding the role of automatic processes in sun protection behavior may therefore also need further attention in future research (Hamilton et al., 2017). However, other determinants, not assessed in this study, could be relevant in predicting sunscreen intentions and behaviors, such as time perspective (preference of short-term over long-term health behavior benefits; Schüz & Eid, 2013) and feelings of autonomy (Pavey & Sparks, 2010). Nevertheless, targeting sociocognitive determinants regarding shade-seeking and clothing intentions and behaviors in future sun safety interventions seems advantageous. Additionally, since the explained variance regarding indirect behaviors was the lowest across behaviors, and parental support and advice are essential in teaching children sun protection behaviors (Dixon et al., 1999; Stanton et al., 2004), an emphasis on indirect behaviors in parental sun safety interventions is recommended. Ideally, both children and their parents should be included in sun safety interventions, since parental behaviors are closely related to children’s own sun protection practices (Dobbinson et al., 2012; Johnson et al., 2001; O’Riordan et al., 2003).

There are a few limitations in this study that are worth mentioning. First, only parental determinants and behaviors were assessed. Although the parental perspective is highly relevant in understanding parent-for-child behaviors (Hamilton et al., 2020), children are increasingly able to perform health behaviors as they grow older (Thoonen et al., 2019) and are therefore important agents in sun safety interventions as well. Future studies could investigate children’s behavioral determinants influencing their own sun protection behaviors. Second, the current study relied on parental self-reports. Although previous studies reveal positive correlations between parental self-reported and objectified sun protection behaviors (Glanz et al., 2009; O’Riordan et al., 2008), future studies could consider objective assessment of behaviors to enhance the validity of our findings. Last, the CIBER approach did not provide the opportunity to examine interactions between determinants and possible confounding factors. CIBER was however carefully selected since the advantages of combining both room for improvement and associated strengths of determinants provide interesting directions for future intervention development. Moreover, we have presented the result stratified by educational level, parental and children’s sex, and children’s age at Open Science Framework (https://osf.io/vwr2g/).

To our knowledge, this study is the first to identify relevance of an extensive set of sociocognitive determinants in predicting direct and indirect parental sun protection behaviors in different situations of sun exposure. The necessity of comprehensive sun safety interventions, targeting specific determinants and behaviors, is evident. In particular, a focus on self-efficacy in future interventions is strongly recommended, using behavior change methods appropriate for this specific determinant (Kok et al., 2016). Enhancement of parental shade-seeking and clothing behaviors seems beneficial since sociocognitive determinants illustrate a vital role in the prediction of these behaviors. Since this study demonstrated strong associations between specific parental determinants and their sun protection intentions and behaviors, composition of future sun safety interventions for children should strongly emphasize the parental role and influence within the family setting.

## Supplemental Material

sj-docx-1-heb-10.1177_10901981211010434 – Supplemental material for Identification of Relevant Sociocognitive Determinants Explaining Multiple Parental Sun Protection Behaviors Click here for additional data file. Supplemental material, sj-docx-1-heb-10.1177_10901981211010434 for Identification of Relevant Sociocognitive Determinants Explaining Multiple Parental Sun Protection Behaviors by Karlijn Thoonen, Liesbeth van Osch, Rik Crutzen, Hein de Vries and Francine Schneider in Health Education & Behavior

## Notes

1. CIBER plots visualizing relevance of sociocognitive determinants stratifying by educational level, parental sex, children’s sex, and children’s age were also generated and can be retrieved from OSF (https://osf.io/vwr2g/)

## References

[bibr1-10901981211010434] Aarts H. Verplanken B. Van Knippenberg A. (1998). Predicting behavior from actions in the past: Repeated decision making or a matter of habit? Journal of Applied Social Psychology, 28(15), 1355–1374. 10.1111/j.1559-1816.1998.tb01681.x

[bibr2-10901981211010434] Ackermann S. Vuadens A. Levi F. Bulliard J.-L. (2016). Sun protective behaviour and sunburn prevalence in primary and secondary schoolchildren in western Switzerland. Swiss Medical Weekly, 146, Article w14370. 10.4414/smw.2016.14370 27878788

[bibr3-10901981211010434] Apalla Z. Lallas A. Sotiriou E. Lazaridou E. Ioannides D. (2017). Epidemiological trends in skin cancer. Dermatology Practical & Conceptual, 7(2), 1. 10.5826/dpc.0702a01 PMC5424654 28515985

[bibr4-10901981211010434] Arnold M. de Vries E. Whiteman D. C. Jemal A. Bray F. Parkin D. M. Soerjomataram I. (2018). Global burden of cutaneous melanoma attributable to ultraviolet radiation in 2012. International Journal of Cancer, 143(6), 1305–1314. 10.1002/ijc.31527 29659012

[bibr5-10901981211010434] Autier P. (2009). Sunscreen abuse for intentional sun exposure. British Journal of Dermatology, 161(s3), 40–45. 10.1111/j.1365-2133.2009.09448.x 19775356

[bibr6-10901981211010434] Autier P. Boniol M. Doré J. F. (2007). Sunscreen use and increased duration of intentional sun exposure: Still a burning issue. International Journal of Cancer, 121(1), 1–5. 10.1002/ijc.22745 17415716

[bibr7-10901981211010434] Bailey R. R. (2019). Goal setting and action planning for health behavior change. American Journal of Lifestyle Medicine, 13(6), 615–618. 10.1177/1559827617729634 31662729 PMC6796229

[bibr8-10901981211010434] Bartholomew Eldredge L. K. Markham C. M. Ruiter R. A. C. Fernández M. E. Kok G. Parcel G. S . (2016). Planning health promotion programs: An intervention mapping approach (4th ed.) Jossey-Bass.

[bibr9-10901981211010434] Behrens C. L. Thorgaard C. Philip A. Bentzen J. (2013). Sunburn in children and adolescents: Associations with parents’ behaviour and attitudes. Scandinavian Journal of Public Health, 41(3), 302–310. 10.1177/1403494813476158 23392996

[bibr10-10901981211010434] Bellamy R. (2005). A systematic review of educational interventions for promoting sun protection knowledge, attitudes and behaviour following the QUESTS approach. Medical teacher, 27(3), 269–275. 10.1080/01421590400029558 16011951

[bibr11-10901981211010434] Bränström R. Ullen H. Brandberg Y. (2004). Attitudes, subjective norms and perception of behavioural control as predictors of sun-related behaviour in Swedish adults. Preventive medicine, 39(5), 992–999. 10.1016/j.ypmed.2004.04.004 15475034

[bibr12-10901981211010434] Bray F. Ferlay J. Soerjomataram I. Siegel R. L. Torre L. A. Jemal A. (2018). Global cancer statistics 2018: GLOBOCAN estimates of incidence and mortality worldwide for 36 cancers in 185 countries. CA: A Cancer Journal for Clinicians, 68(6), 394–424. 10.3322/caac.21492 30207593

[bibr13-10901981211010434] Buller D. B. Borland R. (1999). Skin cancer prevention for children: A critical review. Health Education & Behavior, 26(3), 317–343. 10.1177/109019819902600304 10349571

[bibr14-10901981211010434] Central Committee on Research Involving Human Subjects. (2002). CCMO memorandum behavioural research. https://english.ccmo.nl/investigators/publications/publications/2002/01/01/ccmo-memorandum-behavioural-research

[bibr15-10901981211010434] Cercato M. Nagore E. Ramazzotti V. Sperduti I. Guillén C. (2013). Improving sun-safe knowledge, attitude and behaviour in parents of primary school children: A pilot study. Journal of Cancer Education, 28(1), 151–157. 10.1007/s13187-012-0413-5 23055129

[bibr16-10901981211010434] Champion V. L. Skinner C. S. (2008). The health belief model. In Glanz K. Rimer B. K. Viswanath K. (Eds.), Health behavior and health education: Theory, research, and practice (4th ed., pp. 45–65). Wiley.

[bibr17-10901981211010434] Crutzen R. Peters G.-J. Y. Noijen J. (2017). Using confidence interval-based estimation of relevance to select social-cognitive determinants for behavior change interventions. Frontiers in Public Health, 5, 165. 10.3389/fpubh.2017.00165 28785553 PMC5508122

[bibr18-10901981211010434] Crutzen R. Ygram Peters G.-J. Mondschein C. (2019). Why and how we should care about the General Data Protection Regulation. Psychology & Health, 34(11), 1347–1357. 10.1080/08870446.2019.1606222 31111730

[bibr19-10901981211010434] Day A. K. Stapleton J. L. Natale-Pereira A. M. Goydos J. S. Coups E. J. (2017). Parent and child characteristics associated with child sunburn and sun protection among U.S. Hispanics. Pediatric Dermatology, 34(3), 315–321. 10.1111/pde.13136 28523887

[bibr20-10901981211010434] de Vries H . (2017). An integrated approach for understanding health behavior; the I-change model as an example. Psychology & Behavioral Science International Journal, 2(2), 555–585. 10.19080/pbsij.2.2

[bibr21-10901981211010434] de Vries H. van Osch L. Eijmael K. Smerecnik C. Candel M . (2012). The role of risk perception in explaining parental sunscreen use. Psychology & Health, 27(11), 1342–1358. 10.1080/08870446.2012.684059 22583067

[bibr22-10901981211010434] Dixon H. Borland R. Hill D. (1999). Sun protection and sunburn in primary school children: The influence of age, gender, and coloring. Preventive Medicine, 28(2), 119–130. 10.1006/pmed.1998.0392 10048103

[bibr23-10901981211010434] Dobbinson S. Wakefield M. Hill D. Girgis A. Aitken J. F. Beckmann K. Reeder A. I. Herd N. Spittal M. J. Fairthorne A. Bowles K.-A. (2012). Children’s sun exposure and sun protection: prevalence in Australia and related parental factors. Journal of the American Academy of Dermatology, 66(6), 938–947. 10.1016/j.jaad.2011.06.015 21890234

[bibr24-10901981211010434] Dusza S. W. Halpern A. C. Satagopan J. M. Oliveria S. A. Weinstock M. A. Scope A. Berwick M. Geller A. C. (2012). Prospective study of sunburn and sun behavior patterns during adolescence. Pediatrics, 129(2), 309–317. 10.1542/peds.2011-0104 22271688 PMC3269110

[bibr25-10901981211010434] Gefeller O. Li J. Uter W. Pfahlberg A. B. (2014). The impact of parental knowledge and tanning attitudes on sun protection practice for young children in Germany. International Journal of Environmental Research and Public Health, 11(5), 4768–4781. 10.3390/ijerph110504768 24802677 PMC4053880

[bibr26-10901981211010434] Gefeller O. Uter W. Pfahlberg A. B. (2016). Protection from ultraviolet radiation during childhood: The parental perspective in Bavaria. International Journal of Environmental Research and Public Health, 13(10), 1011. 10.3390/ijerph13101011 PMC5086750 27754448

[bibr27-10901981211010434] Ghiasvand R. Lund E. Edvardsen K. Weiderpass E. Veierød M. (2015). Prevalence and trends of sunscreen use and sunburn among Norwegian women. British Journal of Dermatology, 172(2), 475–483. 10.1111/bjd.13434 25279754

[bibr28-10901981211010434] Glanz K. McCarty F. Nehl E. J. O’Riordan D. L. Gies P. Bundy L. Locke A. E. Hall D. M. (2009). Validity of self-reported sunscreen use by parents, children, and lifeguards. American Journal of Preventive Medicine, 36(1), 63–69. 10.1016/j.amepre.2008.09.012 18945582 PMC2626407

[bibr29-10901981211010434] Glanz K. Rimer B. K. Viswanath K. (2015). Health behavior and health education: Theory, research, and practice (5th ed.). Wiley.

[bibr30-10901981211010434] Gritz E. R. Tripp M. K. James A. S. Carvajal S. C. Harrist R. B. Mueller N. H. Chamberlain R. M. Parcel G. S. (2005). An intervention for parents to promote preschool children’s sun protection: Effects of sun protection is fun! Preventive Medicine, 41(2), 357–366. 10.1016/j.ypmed.2005.01.007 15917033

[bibr31-10901981211010434] Hagger M. S. Hamilton K. (2019). Health behavior, health promotion, and the transition to parenthood: Insights from research in health psychology and behavior change. In Taubman-Ben-Ari O. (Ed.), Pathways and barriers to parenthood (pp. 251–269). Springer. 10.1007/978-3-030-24864-2_15

[bibr32-10901981211010434] Hall H. I. McDavid K. Jorgensen C. M. Kraft J. M. (2001). Factors associated with sunburn in white children aged 6 months to 11 years. American Journal of Preventive Medicine, 20(1), 9–14. 10.1016/S0749-3797(00)00265-8 11137768

[bibr33-10901981211010434] Hamilton K. Cleary C. White K. M. Hawkes A. L. (2016). Keeping kids sun safe: Exploring parents’ beliefs about their young child’s sun-protective behaviours. Psycho-Oncology, 25(2), 158–163. 10.1002/pon.3888 26101815

[bibr34-10901981211010434] Hamilton K. Kirkpatrick A. Rebar A. Hagger M. S. (2017). Child sun safety: Application of an integrated behavior change model. Health Psychology, 36(9), 916. 10.1037/hea0000533 28726470

[bibr35-10901981211010434] Hamilton K. van Dongen A. Hagger M. S. (2020). An extended theory of planned behavior for parent-for-child health behaviors: A meta-analysis. Health Psychology, 39(10), 863. 10.1037/hea0000940 32597678

[bibr36-10901981211010434] Hart K. M. DeMarco R. F. (2008). Primary prevention of skin cancer in children and adolescents: A review of the literature. Journal of Pediatric Oncology Nursing, 25(2), 67–78. 10.1177/1043454208314499 18272782

[bibr37-10901981211010434] Hunter S. Love-Jackson K. Abdulla R. Zhu W. Lee J.-H. Wells K. J. Roetzheim R. (2010). Sun protection at elementary schools: A cluster randomized trial. Journal of the National Cancer Institute, 102(7), 484–492. 10.1093/jnci/djq010 20332388 PMC2902823

[bibr38-10901981211010434] IBM Corp. (2016). IBM SPSS Statistics for Windows. https://hadoop.apache.org

[bibr39-10901981211010434] Jane Pavey L. Sparks P . (2010). Autonomy and reactions to health-risk information. Psychology and Health, 25(7), 855–872. 10.1080/08870440902929528 20204958

[bibr40-10901981211010434] Johnson K. Davy L. Boyett T. Weathers L. Roetzheim R. G. (2001). Sun protection practices for children: Knowledge, attitudes, and parent behaviors. Archives of Pediatrics & Adolescent Medicine, 155(8), 891–896. 10.1001/archpedi.155.8.891 11483115

[bibr41-10901981211010434] Klostermann S. Bolte G. Group G. S. (2014). Determinants of inadequate parental sun protection behaviour in their children: Results of a cross-sectional study in Germany. International Journal of Hygiene and Environmental Health, 217(2-3), 363–369. 10.1016/j.ijheh.2013.07.013 23988730

[bibr42-10901981211010434] Koch S. Pettigrew S. Strickland M. Slevin T. Minto C. (2017). Sunscreen increasingly overshadows alternative sun-protection strategies. Journal of Cancer Education, 32(3), 528–531. 10.1007/s13187-016-0986-5 26792784

[bibr43-10901981211010434] Kok G. Gottlieb N. H. Peters G.-J. Y. Mullen P. D. Parcel G. S. Ruiter R. A. Fernández M. E. Markham C. Bartholomew L. K. (2016). A taxonomy of behaviour change methods: An intervention mapping approach. Health Psychology Review, 10(3), 297–312. 10.1080/17437199.2015.1077155 26262912 PMC4975080

[bibr44-10901981211010434] KWF Kankerbestrijding. (2020). Zonbescherming kinderen [Sun protection for children]. https://www.kwf.nl/kanker-voorkomen/zon-uv-straling-en-huidkanker/zonnetips

[bibr45-10901981211010434] Li J. Uter W. Pfahlberg A. Gefeller O. (2011). Parental perspective on sun protection for young children in Bavaria. Photodermatology, Photoimmunology & Photomedicine, 27(4), 196–202. 10.1111/j.1600-0781.2011.00598.x 21729168

[bibr46-10901981211010434] Linos E. Keiser E. Fu T. Colditz G. Chen S. Tang J. Y. (2011). Hat, shade, long sleeves, or sunscreen? Rethinking U.S. sun protection messages based on their relative effectiveness. Cancer Causes & Control, 22(7), 1067–1071. 10.1007/s10552-011-9780-1 21637987 PMC3873510

[bibr47-10901981211010434] Littlewood Z. Greenfield S. (2018). Parents’ knowledge, attitudes and beliefs regarding sun protection in children: A qualitative study. BMC Public Health, 18(1), Article 207. 10.1186/s12889-018-5091-8 PMC5796497 29391005

[bibr48-10901981211010434] Moinester M. Gottfried R. (2014). Sample size estimation for correlations with pre-specified confidence interval. Quantitative Methods for Psychology, 10(2), 124–130. 10.20982/tqmp.10.2.p0124

[bibr49-10901981211010434] Myers L. B. Horswill M. S. (2006). Social cognitive predictors of sun protection intention and behavior. Behavioral Medicine, 32(2), 57–63. 10.3200/BMED.32.2.57-63 16903615

[bibr50-10901981211010434] Neal D. T. Wood W. Quinn J. M. (2006). Habits: A repeat performance. Current Directions in Psychological Science, 15(4), 198–202. 10.1111/j.1467-8721.2006.00435.x

[bibr51-10901981211010434] Nuffic. (2019). The education system of the Netherlands. https://www.nuffic.nl/sites/default/files/2020-08/education-system-the-netherlands%20%281%29.pdf

[bibr52-10901981211010434] Orbell S. Verplanken B. (2010). The automatic component of habit in health behavior: Habit as cue-contingent automaticity. Health Psychology, 29(4), 374–383. 10.1037/a0019596 20658824

[bibr53-10901981211010434] O’Riordan D. L. Geller A. C. Brooks D. R. Zhang Z. Miller D. R. (2003). Sunburn reduction through parental role modeling and sunscreen vigilance. Journal of Pediatrics, 142(1), 67–72. 10.1067/mpd.2003.mpd039 12520258

[bibr54-10901981211010434] O’Riordan D. L. Glanz K. Gies P. Elliott T. (2008). A pilot study of the validity of self-reported ultraviolet radiation exposure and sun protection practices among lifeguards, parents and children. Photochemistry and Photobiology, 84(3), 774–778. 10.1111/j.1751-1097.2007.00262.x 18179624 PMC3725580

[bibr55-10901981211010434] Peters G.-J. Y. (2018). userfriendlyscience: Quantitative analysis made accessible. https://rdrr.io/github/Matherion/userfriendlyscience/man/userfriendlyscience-package.html https://cran.r-project.org/web/packages/userfriendlyscience/userfriendlyscience.pdf

[bibr56-10901981211010434] Peters G.-J. Y. Crutzen R. (2020). Knowing how effective an intervention, treatment, or manipulation is and increasing replication rates: Accuracy in parameter estimation as a partial solution to the replication crisis. Psychology & Health. 10.1080/08870446.2020.1757098 32378428

[bibr57-10901981211010434] R Core Team. (2017). R: A language and environment for statistical computing. R Foundation for Statistical Computing. https://www.R-project.org/

[bibr58-10901981211010434] Robinson J. K. Rigel D. S. Amonette R. A. (2000). Summertime sun protection used by adults for their children. Journal of the American Academy of Dermatology, 42(5), 746–753. 10.1067/mjd.2000.103984 10775849

[bibr59-10901981211010434] Rodrigue J. R. (1996). Promoting healthier behaviors, attitudes, and beliefs toward sun exposure in parents of young children. Journal of Consulting and Clinical Psychology, 64(6), 1431–1436. 10.1037/0022-006X.64.6.1431 8991330

[bibr60-10901981211010434] Rodrigues A. Sniehotta F. F. Araujo-Soares V. (2013). Are interventions to promote sun-protective behaviors in recreational and tourist settings effective? A systematic review with meta-analysis and moderator analysis. Annals of Behavioral Medicine, 45(2), 224–238. 10.1007/s12160-012-9444-8 23229160

[bibr61-10901981211010434] Sandhu P. K. Elder R. Patel M. Saraiya M. Holman D. M. Perna F. Smith R. A. Buller D. Sinclair C. Reeder A. Makin J. McNoe B. Glanz K. , & the Community Preventive Services Task Force. (2016). Community-wide interventions to prevent skin cancer: Two community guide systematic reviews. American Journal of Preventive Medicine, 51(4), 531–539. 10.1016/j.amepre.2016.03.020 27647053 PMC5031485

[bibr62-10901981211010434] Saraiya M. Glanz K. Briss P. A. Nichols P. White C. Das D. Smith J. Tannor B. Hutchinson A. B. Wilson K. M. Gandhi N. Lee N. C. Rimer B. Coates R. C. Kerner J. F. Hiatt R. A. Buffler P. Rochester P. (2004). Interventions to prevent skin cancer by reducing exposure to ultraviolet radiation: A systematic review. American Journal of Preventive Medicine, 27(5), 422–466. 10.1016/j.amepre.2004.08.009 15556744

[bibr63-10901981211010434] Schüz N. Eid M. (2013). Beyond the usual suspects: Target group-and behavior-specific factors add to a theory-based sun protection intervention for teenagers. Journal of Behavioral Medicine, 36(5), 508–519. 10.1007/s10865-012-9445-x 22790653

[bibr64-10901981211010434] Schwarzer R. (2008). Modeling health behavior change: How to predict and modify the adoption and maintenance of health behaviors. Applied psychology, 57(1), 1–29. 10.1023/A:1013593819121

[bibr65-10901981211010434] Sinclair C. Foley P. (2009). Skin cancer prevention in Australia. British Journal of Dermatology, 161(s3), 116–123. 10.1111/j.1365-2133.2009.09459.x 19775367

[bibr66-10901981211010434] Sniehotta F. F. Scholz U. Schwarzer R. (2005). Bridging the intention–behaviour gap: Planning, self-efficacy, and action control in the adoption and maintenance of physical exercise. Psychol Health, 20(2), 143–160. 10.1080/08870440512331317670

[bibr67-10901981211010434] Stanton W. R. Janda M. Baade P. D. Anderson P. (2004). Primary prevention of skin cancer: A review of sun protection in Australia and internationally. Health Promotion International, 19(3), 369–378. 10.1093/heapro/dah310 15306621

[bibr68-10901981211010434] Statistics Netherlands (CBS). (2016). Standaard Onderwijsindeling 2016 [Standard education classification 2016]. https://www.cbs.nl/nl-nl/onze-diensten/methoden/classificaties/onderwijs-en-beroepen/standaard-onderwijsindeling—soi—/standaard-onderwijsindeling-2021

[bibr69-10901981211010434] Tan M. G. Nag S. Weinstein M. (2018). Parental use of sun protection for their children: Does skin color matter? Pediatric Dermatology, 35(2), 220–224. 10.1111/pde.13433 29436037

[bibr70-10901981211010434] Thomson C. E. White K. M. Hamilton K. (2012). Investigating mothers’ decisions about their child’s sun-protective behaviour using the theory of planned behaviour. Journal of Health Psychology, 17(7), 1001–1010. https://doi.org/10.1177%2F1359105311433905 22253324 10.1177/1359105311433905

[bibr71-10901981211010434] Thoonen K. Schneider F. Candel M. de Vries H. van Osch L. (2019). Childhood sun safety at different ages: Relations between parental sun protection behavior towards their child and children’s own sun protection behavior. BMC Public Health, 19(1), Article 1044. 10.1186/s12889-019-7382-0 PMC6683475 31382940

[bibr72-10901981211010434] TNS-KANTAR. (2019). Privacy policy online survey. https://www.kantar.com/KINA-privacy-policy-survey-research

[bibr73-10901981211010434] Tripp M. K. Diamond P. M. Vernon S. W. Swank P. R. Dolan Mullen P. Gritz E. R. (2013). Measures of parents’ self-efficacy and perceived barriers to children’s sun protection: Construct validity and reliability in melanoma survivors. Health Education Research, 28(5), 828–842. 10.1093/her/cys114 23204537 PMC3858122

[bibr74-10901981211010434] Turner L. R. Mermelstein R. J. (2005). Psychosocial characteristics associated with sun protection practices among parents of young children. Journal of Behavioral Medicine, 28(1), 77–90. 10.1007/s10865-005-2565-9 15887878

[bibr75-10901981211010434] Turrisi R. Hillhouse J. Robinson J. K. Stapleton J. (2007). Mediating variables in a parent based intervention to reduce skin cancer risk in children. Journal of Behavioral Medicine, 30(5), 385–393. 10.1007/s10865-007-9107-6 17453328

[bibr76-10901981211010434] van Osch L. Reubsaet A. Lechner L. Candel M. Mercken L. de Vries H . (2008). Predicting parental sunscreen use: Disentangling the role of action planning in the intention-behavior relationship. Psychology & Health, 23(7), 829–847. 10.1080/08870440701596577 25160883

[bibr77-10901981211010434] van Osch L. Reubsaet A. Lechner L. de Vries H . (2008). The formation of specific action plans can enhance sun protection behavior in motivated parents. Preventive Medicine, 47(1), 127–132. 10.1016/j.ypmed.2008.02.025 18378290

[bibr78-10901981211010434] Von Elm E. Altman D. G. Egger M. Pocock S. J. Gøtzsche P. C. Vandenbroucke J. P . (2007). The Strengthening the Reporting of Observational Studies in Epidemiology (STROBE) statement: Guidelines for reporting observational studies. Annals of Internal Medicine, 147(8), 573–577. 10.1016/j.ijsu.2014.07.013 17938396

[bibr79-10901981211010434] Watson M. Garnett E. Guy G. P. Holman D. M. (2014). The surgeon general’s call to action to prevent skin cancer. U.S. Department of Health & Human Services, Office of the Surgeon General.

[bibr80-10901981211010434] Webb T. L. Sheeran P. (2006). Does changing behavioral intentions engender behavior change? A meta-analysis of the experimental evidence. Psychological Bulletin, 132(2), 249–268. 10.1037/0033-2909.132.2.249 16536643

[bibr81-10901981211010434] White K. M. Starfelt L. C. Young R. M. Hawkes A. L. Cleary C. Leske S. Wihardjo K. (2015). A randomised controlled trial of an online theory-based intervention to improve adult Australians’ sun-protective behaviours. Preventive Medicine, 72(March), 19–22. 10.1016/j.ypmed.2014.12.025 25572618

[bibr82-10901981211010434] Whiteman D. C. Green A. C. Olsen C. M. (2016). The growing burden of invasive melanoma: projections of incidence rates and numbers of new cases in six susceptible populations through 2031. Journal of Investigative Dermatology, 136(6), 1161–1171. 10.1016/j.jid.2016.01.035 26902923

[bibr83-10901981211010434] Whiteman D. C. Whiteman C. A. Green A. C. (2001). Childhood sun exposure as a risk factor for melanoma: A systematic review of epidemiologic studies. Cancer Causes & Control, 12(1), 69–82. 10.1023/A:1008980919928 11227927

[bibr84-10901981211010434] Wu Y. P. Parsons B. G. Aspinwall L. G. Hay J. L. Boucher K. M. Caputo H. Mooney R. Grossman D. Leachman S. A. (2019). Parent and child perspectives on perceived barriers to child sun protection and their association with sun protection strategies among children of melanoma survivors. Pediatric Dermatology, 36(3), 317–323. 10.1111/pde.13796 30895676 PMC6525049

