# Supplementary material for: Identification of Relevant Sociocognitive Determinants Explaining Multiple Parental Sun Protection Behaviors
Source: Health Educ Behav. 2021 May 31;49(3):392–404. doi: 10.1177/10901981211010434 (PMC9150145; doi:10.1177/10901981211010434)
Supplement: sj-docx-1-heb-10.1177_10901981211010434 – Supplemental material for Identification of Relevant Sociocognitive Determinants Explaining Multiple Parental Sun Protection Behaviors [file sj-docx-1-heb-10.1177_10901981211010434.docx]

| **Table 2. Sample distributions and associations with outcome intentions and sun protection behaviors** | | | | | | | | | | | | | | | | | |
| --- | --- | --- | --- | --- | --- | --- | --- | --- | --- | --- | --- | --- | --- | --- | --- | --- | --- |
|  | **Direct** | | | | | | | | | | | | **Indirect** | | | | |
|  | **Sunscreen** | | | | **Clothing** | | | | **Seeking shade** | | | | **Supportive behavior** | | | | |
|  | *Planned* | | *Incidental* | | *Planned* | | *Incidental* | | *Planned* | | *Incidental* | | *Planned* | | *Incidental* | | |
|  | Intention  R^2^= [.54-.65] | Behavior  R^2^=  [.21-.34] | Intention  R^2^=  [.61-.70] | Behavior  R^2^=  [.27-.40] | Intention  R^2^=  [.61-.7] | Behavior  R^2^=  [.32-.44] | Intention  R^2^=  [.66-.74] | Behavior  R^2^=  [.28-.40] | Intention  R^2^=  [.62-.71] | Behavior  R^2^=  [.35-.42] | Intention  R^2^=  [.69-.76] | Behavior  R^2^=  [.37-.49] | Intention  R^2^=  [.42-.54] | Behavior  R^2^=  [.13-.25] | | Intention  R^2^=  [.53-.63] | Behavior  R^2^=  [.22-.35] |
| **Behavior-specific determinants** | | | | | | | | | | | | | | | | | |
| Attitude: importance | r=[0.53; 0.63] | M= [4.49; 4.59], SD=.48, r=[0.32; 0.45] | r=[0.5; 0.61] | M= [4.16; 4.29], SD=.72, r= [0.39; 0.51] | r=[0.57; 0.66] | M= [3.58; 3.72], SD=.83, r=[0.45; 0.56] | r=[0.57; 0.66] | M= [3.64; 3.78], SD=.80, r=[0.39; 0.51] | r=[0.54; 0.64] | M= [3.69; 3.83], SD=.83, r= [0.42; 0.53] | r=[0.59; 0.68] | M= [3.58; 3.73], SD=.89, r= [0.45; 0.56] | r=[0.15; 0.29] | M= [4.23; 4.4], SD=1.18, r= [0.12; 0.27] | | r=[0.28; 0.42] | M=[4.16; 4.31], SD=.91, r=[0.19; 0.34] |
| Attitude: pleasantness | r=[0.3; 0.43] | M= [4.08; 4.23], SD=.94, r=[0.14; 0.29] | r=[0.37; 0.5] | M= [3.9; 4.05], SD=1.00, r= [0.28; 0.41] | r=[0.57; 0.66] | M= [3.32; 3.49], SD=1.15, r=[0.39; 0.51] | r=[0.52; 0.62] | M= [3.52; 3.67], SD=.95, r= [0.35; 0.47] | r=[0.58; 0.67] | M= [3.54; 3.71], SD=1.17, r= [0.41; 0.53] | r=[0.57; 0.66] | M= [3.42; 3.59], SD=1.20, r= [0.43; 0.54] | r=[0.21; 0.35] | M=[4.21; 4.36], SD=.90, r= [0.06; 0.21] | | r=[0.34; 0.47] | M= [4.12; 4.26], SD=.86, r= [0.24; 0.38] |
| Social-norm: partner | r=[0.42; 0.54] | M= [4.47; 4.59], SD=.58, r=[0.21; 0.36] | r=[0.45; 0.57] | M= [4.32; 4.46], SD=.70, r= [0.33; 0.47] | r=[0.53; 0.64] | M= [3.78; 3.94], SD=1.02, r=[0.43; 0.55] | r=[0.51; 0.62] | M= [3.8; 3.96], SD=.98, r= [0.36; 0.49] | r=[0.57; 0.67] | M= [3.72; 3.89], SD=1.10, r= [0.4; 0.53] | r=[0.59; 0.69] | M= [3.65; 3.82], SD=1.15, r= [0.4; 0.53] | r=[0.39; 0.52] | M= [4.23; 4.37], SD=.71, r= [0.13; 0.28] | | r=[0.42; 0.55] | M= [4.15; 4.29], SD=.79, r= [0.31; 0.45] |
| Social-norm: others | r=[0.19; 0.34] | M= [3.96; 4.07], SD=.59, r=[0.12; 0.27] | r=[0.35; 0.47] | M= [3.83; 3.95], SD=.66, r= [0.18; 0.32] | r=[0.44; 0.56] | M= [3.47; 3.6], SD=.69, r=[0.32; 0.45] | r=[0.48; 0.59] | M= [3.43; 3.55], SD=.67, r= [0.28; 0.41] | r=[0.46; 0.57] | M= [3.46; 3.59], SD=.73, r= [0.3; 0.43] | r=[0.48; 0.59] | M= [3.4; 3.53], SD=.72, r= [0.31; 0.44] | r=[0.23; 0.37] | M= [3.74; 3.86], SD=.60, r= [0.02; 0.17] | | r=[0.31; 0.44] | M= [3.65; 3.77], SD=.66, r= [0.09; 0.24] |
| Self-Efficacy: difficulty | r=[0.37; 0.49] | M= [3.78; 3.92], SD=.79, r=[0.24; 0.38] | r=[0.58; 0.67] | M= [3.83; 3.97], SD=.86, r= [0.33; 0.46] | r=[0.5; 0.61] | M= [2.86; 3.02], SD=1.11, r=[0.3; 0.43] | r=[0.61; 0.7] | M= [3.32; 3.47], SD=.97, r= [0.38; 0.5] | r=[0.49; 0.6] | M= [2.66; 2.82], SD=1.19, r= [0.33; 0.46] | r=[0.62; 0.7] | M= [2.82; 2.99], SD=1.2, r= [0.43; 0.55] | r=[0.39; 0.51] | M= [3.51; 3.65], SD=.91, r= [0.16; 0.31] | | r=[0.53; 0.63] | M= [3.53; 3.68], SD=.94, r= [0.24; 0.38] |
| Self-Efficacy: capability | r=[0.61; 0.7] | M= [4.29; 4.41], SD=0.63, r=[0.33; 0.46] | r=[0.66; 0.74] | M= [4.01; 4.15], SD=.85, r= [0.35; 0.48] | r=[0.68; 0.75] | M= [3.19; 3.36], SD=1.23, r=[0.42; 0.53] | r=[0.71; 0.77] | M= [3.42; 3.58], SD=.1.00, r= [0.4; 0.52] | r=[0.67; 0.75] | M= [3.05; 3.22], SD=1.26, r= [0.47; 0.58] | r=[0.72; 0.78] | M= [2.99; 3.16], SD=1.24, r= [0.47; 0.58] | r=[0.55; 0.65] | M= [3.92; 4.06], SD=.80, r= [0.18; 0.32] | | r=[0.63; 0.71] | M= [3.7; 3.85], SD=0.95, r= [0.26; 0.4] |
| Action plans | r=[0.36; 0.49] | M= [3.87; 4.04], SD=1.34,  r=[0.25; 0.39] | r=[0.47; 0.58] | M= [3.66; 3.84], SD=1.45, r= [0.28; 0.41] | r=[0.56; 0.66] | M= [3.05; 3.22], SD=1.37, r=[0.37; 0.5] | r=[0.59; 0.68] | M= [3.18; 3.36], SD=1.35, r= [0.37; 0.5] | r=[0.59; 0.68] | M= [2.82; 3], SD=1.40, r= [0.43; 0.54] | r=[0.6; 0.69] | M= [2.86; 3.03], SD=1.35, r= [0.46; 0.58] | r=[0.41; 0.53] | M= [3.47; 3.65], SD=1.45, r= [0.16; 0.31] | | r=[0.5; 0.6] | M= [3.38; 3.56], SD=1.40, r= [0.24; 0.38] |
| **Generic determinants** | | | | | | | | | | | | | | | | | |
| Knowledge | r=[0.14; 0.29] | M=[4.4; 4.5], SD=.54,  r=[0.07; 0.22] | r=[0.11; 0.26] | r=[0.03; 0.18] | r=[0; 0.15] | r=[-0.02; 0.13] | r=[0.07; 0.22] | r=[0.02; 0.17] | r=[0; 0.15] | r=[0.02; 0.17] | r=[-0.02; 0.13] | r=[-0.02; 0.13] | r=[0.05; 0.2] | r=[0.06; 0.21] | | r=[0.03; 0.18] | r=[0.05; 0.2] |
| R-P: RS of sunburns in PS | r=[0.28; 0.41] | M= [3.87; 4.02] SD=.95, r=[0.19; 0.33] | r=[0.2; 0.34] | r=[0.12; 0.27] | r=[0.08; 0.23] | r=[0.07; 0.22] | r=[0.13; 0.27] | r=[0.05; 0.19] | r=[0.03; 0.18] | r=[0.07; 0.22] | r=[0.02; 0.17] | r=[0.04; 0.19] | r=[0.11; 0.26] | r=[0.05; 0.2] | | r=[0.09; 0.24] | r=[0; 0.15] |
| R-P: Feelings of RS of sunburns in PS | r=[0.31; 0.44] | M= [3.85; 4], SD=1.00, r=[0.23; 0.37] | r=[0.25; 0.39] | r=[0.13; 0.28] | r=[0.09; 0.24] | r=[0.1; 0.25] | r=[0.15; 0.29] | r=[0.08; 0.23] | r=[0.03; 0.18] | r=[0.07; 0.22] | r=[0.02; 0.17] | r=[0.02; 0.17] | r=[0.13; 0.27] | r=[0.05; 0.2] | | r=[0.11; 0.26] | r=[0.03; 0.18] |
| R-P: RS of skin cancer in PS | r=[0.14; 0.28] | M= [3.06; 3.19], SD=.76, r=[0.09; 0.24] | r=[0.16; 0.3] | r=[0.13; 0.27] | r=[0.12; 0.26] | r=[0.12; 0.27] | r=[0.13; 0.27] | r=[0.03; 0.18] | r=[0.14; 0.28] | r=[0.08; 0.23] | r=[0.12; 0.27] | r=[0.05; 0.2] | r=[0.12; 0.27] | r=[-0.01; 0.14] | | r=[0.12; 0.26] | r=[0.01; 0.16] |
| R-P: Feelings of RS of skin cancer in PS | r=[0.15; 0.29] | M= [2.98; 3.12], SD=.82, r=[0.13; 0.27] | r=[0.21; 0.35] | r=[0.19; 0.33] | r=[0.15; 0.29] | r=[0.16; 0.31] | r=[0.16; 0.31] | r=[0.09; 0.23] | r=[0.13; 0.27] | r=[0.10; 0.25] | r=[0.12; 0.27] | r=[0.08; 0.23] | r=[0.12; 0.26] | r=[0.03; 0.19] | | r=[0.13; 0.27] | r=[0.09; 0.24] |
| R-P: RS of sunburns in IS | r=[0.18; 0.32] | M= [3.29; 3.44], SD=.98, r=[0.10; 0.25] | r=[0.23; 0.36] | r=[0.21; 0.35] | r=[0.11; 0.26] | r=[0.11; 0.26] | r=[0.16; 0.31] | r=[0.1; 0.25] | r=[0.13; 0.28] | r=[0.15; 0.29] | r=[0.18; 0.33] | r=[0.16; 0.3] | r=[0.07; 0.22] | r=[0.01; 0.16] | | r=[0.11; 0.25] | r=[0.08; 0.23] |
| Feelings of RS: sunburns in IS | r=[0.16; 0.3] | M= [3.22; 3.37], SD=1.10, r=[0.13; 0.28] | r=[0.30; 0.43] | r=[0.27; 0.4] | r=[0.16; 0.3] | r=[0.14; 0.28] | r=[0.19; 0.33] | r=[0.13; 0.27] | r=[0.14; 0.29] | r=[0.16; 0.3] | r=[0.18; 0.32] | r=[0.16; 0.31] | r=[0.06; 0.21] | r=[0.03; 0.19] | | r=[0.16; 0.3] | r=[0.14; 0.29] |
| R-P: RS of skin cancer in IS | r=[0.09; 0.24] | M= [2.84; 2.98], SD=.82, r=[0.07; 0.22] | r=[0.15; 0.29] | r=[0.19; 0.33] | r=[0.10; 0.25] | r=[0.09; 0.24] | r=[0.11; 0.25] | r=[0.04; 0.19] | r=[0.14; 0.28] | r=[0.11; 0.25] | r=[0.16; 0.31] | r=[0.12; 0.27] | r=[0.05; 0.2] | r=[-0.03; 0.13] | | r=[0.11; 0.26] | r=[0.05; 0.2] |
| R-P: Feelings of RS of skin cancer in IS | r=[0.07; 0.22] | M= [2.79; 2.93], SD=.84, r=[0.06; 0.21] | r=[0.17; 0.31] | r=[0.22; 0.36] | r=[0.14; 0.28] | r=[0.14; 0.28] | r=[0.14; 0.28] | r=[0.08; 0.23] | r=[0.15; 0.29] | r=[0.14; 0.29] | r=[0.18; 0.32] | r=[0.16; 0.3] | r=[0.02; 0.17] | r=[0.01; 0.16] | | r=[0.09; 0.24] | r=[0.12; 0.27] |
| R-P: severity of skin cancer | r=[0.14; 0.28] | M= [3.79; 3.92], SD=.78, r=[0.11; 0.25] | r=[0.20; 0.34] | r=[0.11; 0.26] | r=[0.10; 0.24] | r=[0.03; 0.18] | r=[0.11; 0.26] | r=[-0.03; 0.12] | r=[0.06; 0.21] | r=[0; 0.15] | r=[0.07; 0.22] | r=[0.01; 0.16] | r=[0.1; 0.25] | r=[0.04; 0.2] | | r=[0.12; 0.26] | r=[0.07; 0.22] |
| R-P: Severity compared to other cancer types | r=[0.04; 0.19] | M= [3.39; 3.54], SD=.91, r=[0.05; 0.2] | r=[0.11; 0.26] | r=[0.10; 0.25] | r=[0.14; 0.28] | r=[0.04; 0.19] | r=[0.10; 0.25] | r=[-0.02; 0.13] | r=[0.11; 0.25] | r=[0; 0.15] | r=[0.12; 0.26] | r=[0.05; 0.2] | r=[0.09; 0.23] | r=[0.05; 0.21] | | r=[0.11; 0.25] | r=[0.09; 0.24] |
| R-P: Severity compared to other diseases | r=[0.11; 0.26] | M= [3.58; 3.73], SD=.91, r=[0.07; 0.21] | r=[0.17; 0.31] | r=[0.13; 0.27] | r=[0.15; 0.29] | r=[0.05; 0.2] | r=[0.14; 0.29] | r=[-0.03; 0.12] | r=[0.14; 0.28] | r=[0.02; 0.17] | r=[0.15; 0.29] | r=[0.02; 0.17] | r=[0.11; 0.25] | r=[0.01; 0.17] | | r=[0.12; 0.27] | r=[0.07; 0.22] |
| R-P: Severe consequences for the child | r=[0.12; 0.27] | M= [3.8; 3.94], SD=.84, r=[0.08; 0.23] | r=[0.17; 0.31] | r=[0.13; 0.27] | r=[0.06; 0.21] | r=[0.02; 0.17] | r=[0.05; 0.2] | r=[-0.08; 0.08] | r=[0.05; 0.2] | r=[0.02; 0.17] | r=[0.07; 0.22] | r=[0; 0.15] | r=[0.06; 0.21] | r=[0.02; 0.17] | | r=[0.08; 0.22] | r=[0.07; 0.23] |
| AR: feelings of regret, sunburns | r=[0.16; 0.31] | M= [3.87; 4.03], SD=1.00, r=[0.12; 0.27] | r=[0.26; 0.39] | r=[0.22; 0.36] | r=[0.18; 0.32] | r=[0.16; 0.3] | r=[0.17; 0.31] | r=[0.07; 0.22] | r=[0.16; 0.3] | r=[0.14; 0.28] | r=[0.16; 0.3] | r=[0.12; 0.26] | r=[0.17; 0.31] | r=[0.08; 0.24] | | r=[0.22; 0.36] | r=[0.13; 0.28] |
| AR: feelings of regret, skin cancer | r=[0.22; 0.36] | M= [4.48; 4.6], SD=.65, r=[0.11; 0.26] | r=[0.25; 0.38] | r=[0.14; 0.29] | r=[0.06; 0.21] | r=[0.04; 0.19] | r=[0.11; 0.26] | r=[0; 0.15] | r=[0.01; 0.16] | r=[0.02; 0.17] | r=[0.02; 0.17] | r=[-0.02; 0.13] | r=[0.14; 0.28] | r=[0.02; 0.18] | | r=[0.13; 0.28] | r=[0.02; 0.17] |
| AR: occurrence of sunburns | r=[0.12; 0.26] | M= [3.86; 4.02], SD=1.05, r=[0.13; 0.28] | r=[0.20; 0.34] | r=[0.22; 0.36] | r=[0.16; 0.31] | r=[0.15; 0.3] | r=[0.14; 0.29] | r=[0.08; 0.23] | r=[0.14; 0.28] | r=[0.17; 0.31] | r=[0.15; 0.3] | r=[0.14; 0.28] | r=[0.13; 0.27] | r=[0.05; 0.2] | | r=[0.19; 0.33] | r=[0.13; 0.28] |
| AR: development of skin cancer | r=[0.19; 0.33] | M= [4.51; 4.62], SD=.57, r=[0.11; 0.26] | r=[0.22; 0.36] | r=[0.16; 0.3] | r=[0.04; 0.19] | r=[0.05; 0.2] | r=[0.07; 0.22] | r=[0.03; 0.18] | r=[0.01; 0.16] | r=[0.06; 0.21] | r=[0.03; 0.18] | r=[0.03; 0.18] | r=[0.09; 0.24] | r=[0.01; 0.16] | | r=[0.10; 0.25] | r=[0.02; 0.17] |
| CtA: sunburns previous summer | r=[-0.12; 0.03] | M= [1.29; 1.37], SD=.26, r=[-0.20; -0.05] | r=[-0.15; 0] | r=[-0.22; -0.07] | r=[-0.16; -0.01] | r=[-0.17; -0.02] | r=[-0.16; -0.01] | r=[-0.18; -0.03] | r=[-0.11; 0.04] | r=[-0.16; -0.01] | r=[-0.15; 0] | r=[-0.12; 0.03] | r=[-0.12; 0.03] | r=[-0.10; 0.05] | | r=[-0.15; 0] | r=[-0.11; 0.05] |
| CtA: sunburns lifetime | r=[-0.10; 0.05] | M= [1.77; 1.86], SD=.41, r=[-0.13; 0.02] | r=[-0.13; 0.02] | r=[-0.17; -0.02] | r=[-0.21; -0.06] | r=[-0.21; -0.06] | r=[-0.19; -0.04] | r=[-0.2; -0.05] | r=[-0.22; -0.07] | r=[-0.25; -0.11] | r=[-0.23; -0.08] | r=[-0.24; -0.09] | r=[-0.12; 0.03] | r=[-0.07; 0.09] | | r=[-0.16; -0.01] | r=[-0.12; 0.04] |
| Attitude child’s tanned skin | r=[-0.34; -0.2] | M= [1.78; 1.93], SD=.91, r=[-0.27; -0.13] | r=[-0.37; -0.23] | r=[-0.31; -0.17] | r=[-0.23; -0.08] | r=[-0.24; -0.09] | r=[-0.28; -0.13] | r=[-0.24; -0.09] | r=[-0.26; -0.11] | r=[-0.24; -0.09] | r=[-0.23; -0.08] | r=[-0.22; -0.08] | r=[-0.25; -0.1] | r=[-0.24; -0.09] | | r=[-0.34; -0.2] | r=[-0.28; -0.13] |

Footnote: PS = planned sun exposure situations, IS = incidental sun exposure situations, R-P = Risk-perception, RS = Risk susceptibility, AR = Anticipated regret, CtA = Cue to Action, r= CI’s of associations, M=CI’s of sample means, SD=Standard Deviation of the sample means
